# Bilateral facial colliculus syndrome caused by pontine tegmentum infarction: a case report

**DOI:** 10.1186/s12883-021-02524-x

**Published:** 2021-12-20

**Authors:** Sheng Zhuang, Weiye Xie, Chengjie Mao

**Affiliations:** Department of Neurology and Suzhou Clinical Research Center of Neurological Disease, The Second Affiliated Hospital of Soochow University, 1055 Sanxiang Road, Suzhou, 215000 China

**Keywords:** Pontine tegmentum, Patent foramen ovale, Eye movement

## Abstract

**Background:**

Bilateral facial colliculus syndrome is a rare clinical presentation in patient with pontine infarction. We herein described a case of bilateral facial paralysis and complete horizontal gaze palsy possibly caused by paradoxical embolization from patent foramen ovale related stroke.

**Case presentation:**

A 55-year-old male presented with sudden onset of complete peripheral facial palsy and horizontal gaze palsy after Valsava maneuver. MRI revealed symmetric involvement of bilateral pontine tegmentum in accordance with the location of facial colliculus. CSF analysis and follow-up MRI showed no evidence of central demyelinating disease. Subsequent echocardiography revealed patent foramen ovale and closure surgery was performed.

**Conclusions:**

Facial colliculus syndrome with symmetric dorsal pontine tegmentum involvement may a rare manifestation in posterior circulation stroke.

## Background

Facial colliculus syndrome is an uncommon manifestation featured by horizontal gaze palsy and peripheral facial nerve palsy, which is rarely encountered in clinical practice with differential diagnosis including demyelinating disease (e.g. multiple sclerosis) or cerebrovascular event (e.g infarction and hemorrhage). Bilateral facial colliculus syndrome with symmetric involvement of the pontine tegmentum potentially caused by patent foramen ovale related stroke was not reported previously. We herein described a unique case of paradoxical embolization presenting as bilateral facial colliculus syndrome.

## Case presentation

**Fig. 1:**
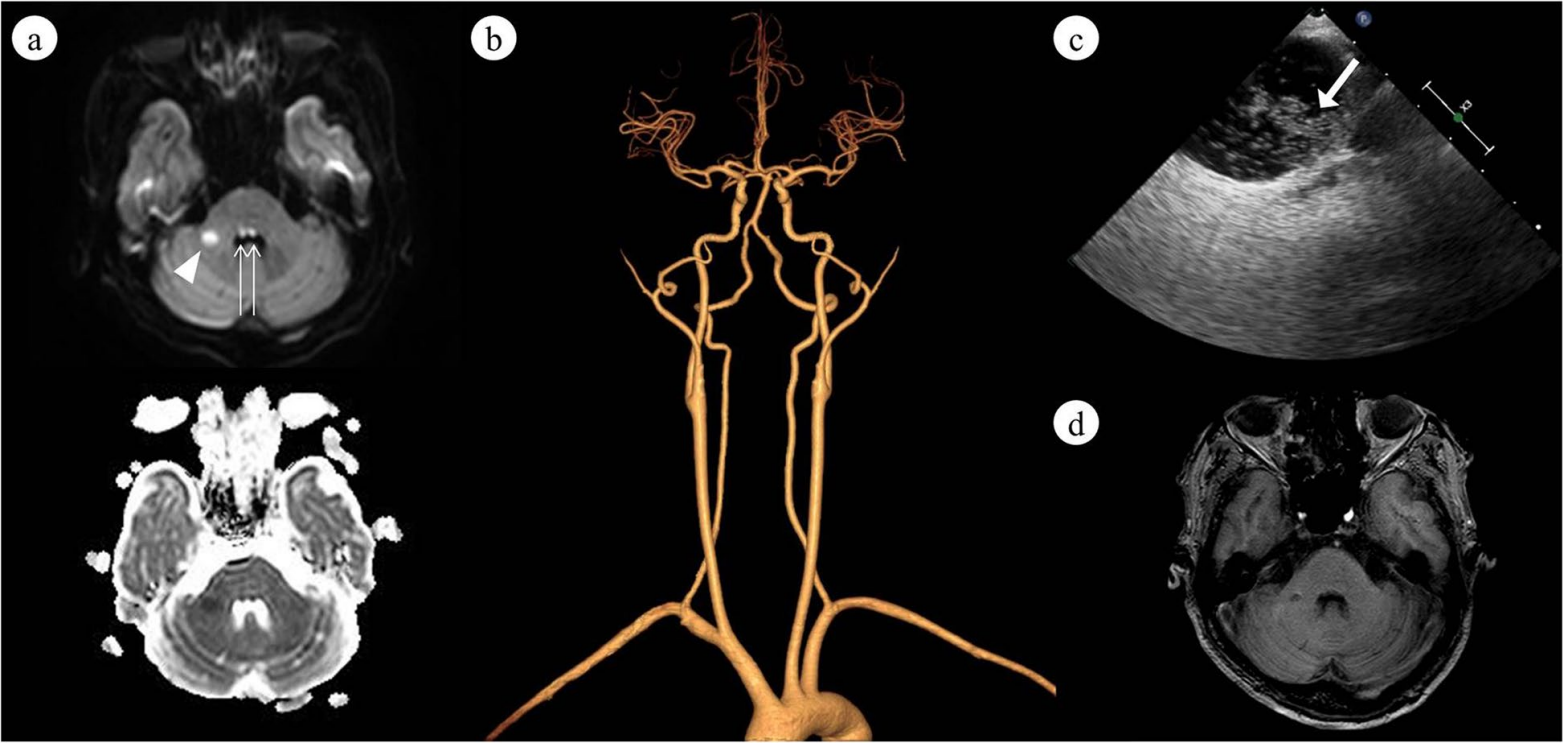
**a** Diffusion-weighted image 1 day after disease onset revealed increased restricted diffusion signal in the right middle cerebellar peduncle (arrowhead) and bilateral dorsal pontine tegmentum (thin arrows), the latter of which was consistent with the involvement of facial colliculus. The lesions showed correspondent hyposignal on attenuated diffusion coefficient map. **b** CT angiography of head and neck did not show obvious stenosis in the anterior and posterior circulation. **c** Right-to-left mircobubbles (thick arrow) were observed on contrast-enhanced transoesophageal echocardiography during Valsalva maneuver. **d** Follow-up MRI at 6 month showed hypointenstiy in the corresponding region on T1 weighted image, indicating old infarction

A 55-year-old man presented with sudden onset of dizziness, diplopia, difficulty of closing eyes, and trouble of chewing after lifting heavy goods 3 days prior to admission. He had a history of mild hypertension for 1 year with treatment of amlodipine 2.5 mg/d and poorly-controlled asthma. On examination, he showed completed bilateral horizontal gaze palsy which was uncorrected by vestibuloocular reflex. Gazed-evoked upbeat nystagmus (UBN) was observed on attempted upward gaze but not on straight-ahead gaze position. In addition, he had bilateral peripheral facial paralysis with predominance on the left. Examination on other cranial nerves, including facial sensation, taste, hearing, and pharyngeal reflex, were normal. Mild ataxia was noticed on the left upper extremity when performing finger-to-nose test. His muscle strength was 5 on four limbs and he had normal pinprick sensation and brisk tendon reflexes. Brain MRI revealed hyperintensity in right middle cerebellar peduncle and bilateral dorsal pontine tegmentum on diffusion-weighted image, indicating new infarction (Fig. 1a). No periventricular white matter lesions were observed. CT angiography of vertebrobasilar artery showed no evidence of significant stenosis (Fig. 1b). CSF examination showed normal protein level and CSF analysis for oligoclonal band, myelin oligodendrocyte glycoprotein antibody, and aquaporin-4 antibody were negative. Contrast-enhanced transcranial doppler showed > 50 microbubbles during the Valsava maneuver, suggesting potential cardiac right-to-left shunt. Further transoesophageal echocardiography revealed patent foramen ovale (PFO; Fig. 1c) with a tunnel length of 12.6 mm. Transthoracic echocardiography revealed left atrial diameter of 32 mm and left ventricular ejection fraction of 70.4%. No left ventricle hypertrophy or atrial septal aneurysm was observed. Holter monitor examination in hospital did not capture remarkable arrhythmias, i.e. atrial flutter or atrial fibrillation. Rrivaroxaban 15 mg/d was prescribed at discharge and closure of PFO was then administrated. His symptoms relieved and follow-up MRI at 6 month showed an old infarction of the same region on T1 weighted image (Fig. 1d) without other evidence of demyelination.

## Discussion

Lesion of the sixth nerve nucleus or paramedian pontine reticular formation and the adjacent medial longitudinal fasciculus (MLF) forms the classic manifestation of one-and-a-half syndrome, that is, ipsilateral gaze palsy and internuclear ophthalmoplegia (INO). On its basis, an additional involvement of seventh nerve causing peripheral facial palsy was called eight-and-a-half syndrome [1]. Because of the close proximity of these structures, lesions in dorsal pontine tegmentum may lead to several variants of eight-and-a-half syndrome [2]. In our case, the patient showed bilateral eight-and-a-half syndrome (also known as the 16-syndrome [3–5]) with radiological evidence of symmetric caudal pontine involvement. Precisely, the lesion of current case was located in bilateral facial colliculus, an elevation on the floor of the fourth ventricle in the dorsal pons housing the abducens nucleus (contains abducens nerve and MLF) and genu of facial nerve. Therefore, in our patient, we speculate that the complete ophthalmoplegia on horizontal gaze was bilateral INO resulting from the abducens nucleus involvement and the facial palsy was from the involvement of both facial nerves at the genu. In our case, the action of lifting goods, similar to the Valsava maneuver, might aggravate right-to-left shunt thus contributing to potential embolism in the distal part of paramedian potine perforating artery with its terminal supplying both sides of dorsal pons.

Impairment of MLF may be responsible for UBN as the structure affects vertical eye velocity in both directions but slightly more for the downward system, resulting in compensating UBN [6]. Moreover, bilateral MLF lesions causing INO was reported to be more frequent in upward gaze-evoked nystagmus but not on primary gaze position [7–9], which was consistent with our case. The occurrence of UBN was also related with damage to the ventral tegmental tract (VTT) arising from the superior vestibular nucleus [6]. However, pontine damage involving VTT are mostly large lesions in the ventral tegmentum or the posterior basis pontis at the upper pons level [6], which was different from the restricted small lesions of dorsal pontine in our patient. Therefore, the gazed-evoked UBN in our case was more likely to result from MLF involvement rather than VTT involvement.

With respect to differential diagnosis, symmetric dorsal pontine tegmentum lesions should raise the awareness of demyelinating disease such as multiple sclerosis [3] or neuromyelitis optica spectrum disorders. In our case, a rapid onset to the disease peak during Valsava maneuver, negative finding of demyelination evidences in CSF and follow-up MRI, and improvement of symptoms after stroke intervention add support to the diagnosis of a pontine tegmentum infarction.

## Conclusion

Bilateral facial colliculus syndrome is a rare clinical presentation of pontine infarction apart from demyelinating disease.

## Data Availability

Data sharing is not applicable to this article as no datasets were generated or analysed during the current study.

## Abbreviations

UBN: Gazed-evoked upbeat nystagmus
CT: Computed tomography
MRI: Magnetic resonance imaging
CSF: Cerebrospinal fluid
PFO: Patent foramen ovale
MLF: Medial longitudinal fasciculus
INO: Internuclear ophthalmoplegia
VTT: Ventral tegmental tract
